# Entrance dose measurements for *in‐vivo* diode dosimetry: Comparison of correction factors for two types of commercial silicon diode detectors

**DOI:** 10.1120/jacmp.v1i3.2642

**Published:** 2000-06-01

**Authors:** X. R. Zhu

**Affiliations:** ^1^ Department of Radiation Oncology Medical College of Wisconsin 9200 West Wisconsin Avenue Milwaukee Wisconsin 53226

**Keywords:** *in‐vivo* dosimetry, diode detector

## Abstract

Silicon diode dosimeters have been used routinely for *in‐vivo* dosimetry. Despite their popularity, an appropriate implementation of an *in‐vivo* dosimetry program using diode detectors remains a challenge for clinical physicists. One common approach is to relate the diode readout to the entrance dose, that is, dose to the reference depth of maximum dose such as $d_{\max}$ for the $10\times 10\;{\text{cm}}^{2}$ field. Various correction factors are needed in order to properly infer the entrance dose from the diode readout, depending on field sizes, target‐to‐surface distances (TSD), and accessories (such as wedges and compensate filters). In some clinical practices, however, no correction factor is used. In this case, a diode‐dosimeter‐based *in‐vivo* dosimetry program may not serve the purpose effectively; that is, to provide an overall check of the dosimetry procedure. In this paper, we provide a formula to relate the diode readout to the entrance dose. Correction factors for TSD, field size, and wedges used in this formula are also clearly defined. Two types of commercial diode detectors, ISORAD (*n*‐type) and the newly available QED (*p*‐type) (Sun Nuclear Corporation), are studied. We compared correction factors for TSDs, field sizes, and wedges. Our results are consistent with the theory of radiation damage of silicon diodes. Radiation damage has been shown to be more serious for *n*‐type than for *p*‐type detectors. In general, both types of diode dosimeters require correction factors depending on beam energy, TSD, field size, and wedge. The magnitudes of corrections for QED (*p*‐type) diodes are smaller than ISORAD detectors.

PACS number(s): 87.66.–a, 87.52.–g

## INTRODUCTION

The outcome of radiation therapy may depend upon tumor doses that do not vary by more than ±5% about the optimum.^1^ *In‐vivo* dosimetry provides an overall check of the entire dosimetry procedure and patient setup.^2^ Using silicon diode dosimeters for *in‐vivo* dosimetry has become common practice in radiation oncology in recent years.^3^ ^–^ ^13^ The advantages of using diode detectors for patient dosimetry include small size, no bias, and immediate readout. Such an *in‐vivo* dose measurement can be performed as entrance dose only and entrance and exit dose determinations.^2^ ^–^ ^11^ The entrance dose only *in‐vivo* dosimetry program can effectively provide an overall check of the dosimetry and treatment delivery processes, including monitoring unit calculations, patient setup, and proper treatment accessories and field size being used.^9^ With entrance and exit dose measurements, additional errors, including changes in patient thickness, contouring errors, and problems with CT data transfer, can be detected.^3^ ^,^ ^7^ ^,^ ^9^ In our clinical practices, diode detectors have been used for measuring entrance doses only for treatment quality assurance. For an ideal detector, the entrance dose (maximum dose, $D_{\max}$, in cGy) can be directly derived from reading a calibrated diode. In reality, however, depending on field sizes (FS), target‐to‐patient‐surface distance (TSD), and accessories (such as wedges), it is necessary to apply various correction factors (CFs) to a diode reading in order to infer $D_{\text{entrance}}$.^3^ ^,^ ^5^ ^–^ ^10^ In this paper, we compared the TSD, field size, and wedge dependence of two types: ISORAD (*n*‐type) and the newly available QED (*p*‐type) commercial diode detectors.

## MATERIALS AND METHODS

### Diode readings and correction factors

In this section, we define all the parameters used in our *in‐vivo* diode dosimetry program. We relate diode readings to maximum dose, that is, entrance dose. Let us assume that the diode dosimeter is placed on the surface of a flat phantom with TSD equal to ${\text{TCD}}_{\text{diode}}$ for calibration, where the TCD diode stands for target to calibration distance for the diode. The diode reading equals to ${\text{RDG}}_{\text{DC}}$ for 100 monitor units (MU) delivered under this calibration condition. Then, for a diode placed on the skin of the patient with target‐to‐skin distance equal to TSD, the diode reading (RDG) is given by

(1) $$\text{RDG}=\frac{{\text{RDG}}_{\text{DC}}}{100}\times \text{MU}\times {\text{OF}}_{\text{diode}}\times {\text{AF}}_{\text{diode}}\times {\left(\frac{{\text{TCD}}_{\text{diode}}}{\text{TSD}}\right)}^{2}\times \frac{1}{{\text{CF}}_{\text{TSD}}},$$

where MU is the monitor units delivered for the field under consideration; ${\text{OF}}_{\text{diode}}$ is the output factor of the diode which is defined as the ratio of the diode readout for the current field to the readout for the reference field size of $10\times 10\;{\text{cm}}^{2}$; ${\text{RDG}}_{\text{FS}}/{\text{RDG}}_{10\times 10}$, ${\text{AF}}_{\text{diode}}$ is the accessory (such as wedge) factor of the diode which is equal to the ratio of the diode readout with the accessory in the beam to the readout without the accessory, ${\text{RDG}}_{\text{accessory}}/{\text{RDG}}_{\text{open}}$; and ${\text{CF}}_{\text{TSD}}$ is the correction factor accounting for deviation of diode readings from the inverse square law, and is given by

$${\text{CF}}_{\text{TSD}}={\left(\frac{{\text{TCD}}_{\text{diode}}}{\text{TSD}}\right)}^{2}/\left(\frac{{\text{RDG}}_{\text{TSD}}}{{\text{RDG}}_{{\text{TCD}}_{\text{diode}}}}\right),$$

where ${\text{RDG}}_{{\text{TCD}}_{\text{dilode}}}$ and ${\text{RDG}}_{\text{TSD}}$ are diode readings for the field size of $10\times 10\;{\text{cm}}^{2}$ at ${\text{TCD}}_{\text{diode}}$ for diode calibration and at TSD, respectively. Here, we have assumed that ${\text{CF}}_{\text{TSD}}$ is independent of field size and accessories. For a patient treated with a photon beam with a collimator size of $r_{c}$, the equivalent field size of $r_{d}$ at a depth of *d* and dose to target of TD, the MU is given by

(2) $$\text{MU}=\frac{\text{TD}}{\text{TMR}(d,r_{d})\times S_{c}(r_{c})\times S_{p}(r_{d})\times \text{AF}\times {\left(\frac{\text{TCD}}{\text{TSD}+d}\right)}^{2}},$$

where $\text{TMR}(d,r_{d})$ is the tissue maximum ratio, $S_{c}(r_{c})$ is the collimator scatter factor, $S_{p}(r_{d})$ is the phantom scatter factor, AF is the accessory factor, and TCD is the distance from target to the calibration point of the photon beam. Here we have assumed that photon beams are calibrated to 1.00 cGy/MU for the reference field size at TCD. Combining Eqs. (1) and (2) we have

(3) $$D_{\text{entrance}}={\text{RDG}}_{\text{Corr}}\times \frac{100}{{\text{RDG}}_{\text{DC}}}\times {\left(\frac{\text{TCD}}{{\text{TCD}}_{\text{diode}}}\right)}^{2}\times {\left(\frac{\text{TSD}}{\text{TSD}+d_{\max}}\right)}^{2},$$

where ${\text{RDG}}_{\text{Corr}}=\text{RDG}\times {\text{CF}}_{\text{TSD}}\times {\text{CF}}_{\text{FS}}\times {\text{CF}}_{\text{accessory}}$ is the corrected diode reading; ${\text{CF}}_{\text{FS}}=S_{c,p}/{\text{OF}}_{\text{diode}}$ is the correction factor for the diode output factor, where $S_{c,p}=S_{c}S_{p}$ is the total scatter factor; ${\text{CF}}_{\text{accessory}}=\text{AF}/{\text{AF}}_{\text{diode}}$ is the correction factor for the diode accessory factor; and $D_{\text{entrance}}$ is the measured entrance dose based on diode readout. The measured entrance dose is then compared with the calculated $D_{\max}=(\text{TD}/\text{TMR})\times {\left[(\text{TSD}+d)/(\text{TSD}+d_{\max})\right]}^{2}$. For a patient treated with a TSD setup, it can be shown that Eq. (3) is still valid, but $D_{\max}=\text{TD}\times 100/\text{PDD}$, where PDD is the percentage depth dose. For an ideal diode dosimeter, all correction factors defined above are equal to 1.

Equations similar to Eq. (3) have been used by various authors to relate to diode readings to entrance doses and to define correction factors.^5^ ^–^ ^10^ The last three factors in Eq. (3) are often lumped together into a single factor called the calibration factor, $F_{\text{calib}}$, by these authors. Our approach provides some insight into the calibration factor, which depends on calibration conditions for both the photon beam and the diode dosimeter, beam quality $\left(d_{\max}\right)$, and TSD for the field under treatment. The first term, $100/{\text{RDF}}_{\text{DC}}$, depends only on the readout of the diode under the calibration condition. The first inverse square term in Eq. (3) is due to the fact that photon beam is calibrated at TCD, while the diode is calibrated at ${\text{TCD}}_{\text{diode}}$, which may not be the same. The second inverse square term exists because the diode is placed on the patient's skin, while entrance dose refers to dose at $d_{\max}$. Two examples are given below for $d_{\max}=3\;\text{cm}$, ${\text{TCD}}_{\text{diode}}=100\;\text{cm}$, and ${\text{RDG}}_{\text{DC}}=100$. First, a photon beam is calibrated with the isocentric setup (i.e., 1 cGy/MU at 100 cm from the target in this case), when TSD=100 cm, $D_{\text{entrance}}=0.943\times {\text{RDG}}_{\text{Corr}}$; when TSD=70 cm, $D_{\text{entrance}}=0.919\times {\text{RDG}}_{\text{Corr}}$; and when TSD=130 cm, $D_{entrance}=0.955\times {\text{RDG}}_{\text{corr}}$. Second, for a photon beam calibrated with the TSD setup (i.e., 1 cGy/MU at 103 cm from the target for this example), when TSD=100 cm, $D_{\text{entrance}}={\text{RDG}}_{\text{corr}}$; when TSD=70 cm, $D_{\text{entrance}}=0.976\times {\text{RDG}}_{\text{Corr}}$; and when TSD=130 cm, $D_{\text{entrance}}=1.013\times {\text{RDG}}_{\text{Corr}}$. It is clearly demonstrated that the calibration factor depends on the calibration setups for the photon beam itself and the diode detector as well as TSD of the treatment field. The calibration factor, $F_{\text{calib}}$, can significantly deviate from 1.00 as illustrated in the above examples. To force $F_{\text{calib}}$ close to 1.00, one could choose the diode calibration distance, ${\text{TCD}}_{\text{diode}}$, equal to $\text{TCD}-d_{\max}$, regardless of how the photon beam was calibrated. If this approach was used, the inverse square factors in Eq. (3) would be within 1.00±0.03 for an 18‐MV photon beam with $d_{\max}=3\;\text{cm}$, as shown in the above example for the photon beam calibrated with the TSD setup.

### Diode detectors and measurements

A total of four diode dosimeters were investigated, two for ISORAD (*n*‐type) and two for QED (*p*‐type) diodes (Sun Nuclear). Among ISORAD and QED diodes, one is for 6‐MV and one is for 18‐MV x rays. The details of cross‐section drawings of ISORAD and QED diode detectors are available from the vendor.

There are three noticeable differences between ISORAD and QED diode detectors. First, *n*‐type silicon is used by ISORADs, while *p*‐type silicon is used for QEDs. The second difference is the packaging of the diode detectors—ISORADs are cylindrical in shape, while QEDs have a flat bottom and hemispherical shape. The third difference is that ISORAD diodes use brass as a buildup material $\left(1.53\;g/{\text{cm}}^{2}\right)$ for 6–12‐MV detectors and tungsten for 15–25‐MV detectors $\left(2.75\;g/{\text{cm}}^{2}\right)$, while QED diodes use $1.85\;g/{\text{cm}}^{2}$ brass for 6–12‐MV detectors, and $3.04\;g/{\text{cm}}^{2}$ brass for 15–25‐MV dosimeters.

Photon beams with nominal energies of 6 and 18 MV from a linear accelerator (VARIAN CL2100C) were calibrated using the TSD setup according to AAPM TG–21 protocol.^14^ The calibrated photon beams deliver 1.00 cGy/MU in water for the $10\times 10\;{\text{cm}}^{2}$ square field at the distance of $100+d_{\max}\;\text{cm}$ from the target. Diode detectors for *in‐vivo* dosimetry were calibrated at the TSD of 100 cm for the field size of $10\times 10\;{\text{cm}}^{2}$. Under this calibration condition, a calibrated diode dosimeter would have a reading that is numerically equal to the number of MU delivered.

Diode detectors were placed atop a polystyrene phantom for all measurements determining CFs. We measured the dependence of diode readings on TSD for the collimator setting of $10\times 10\;{\text{cm}}^{2}$, on field sizes at TSD of 100 cm and on wedges at TSD of 100 cm and the field size of $10\times 10\;{\text{cm}}^{2}$. Wedges discussed here are so‐called “upper wedges,” normally used in combination with multileaf collimator for Varian linear accelerators. All correction factors were then calculated according to the definitions given in Eqs. (1) and (3).

Total scatter factors, $S_{c,p}$, were measured by a Farmer‐type ion chamber in a water phantom at the reference depth (that is, depth of maximum dose for the field size of $10\times 10\;{\text{cm}}^{2}$ at TSD of 100 cm), while collimator scatter factors, $S_{c}$, were measured with an ion chamber with a buildup cap (a Lucite cap for 6 MV and an aluminum one for 18 MV) at the isocenter.

## RESULTS

### TSD correction factors

Table I shows the correction factors as a function of TSD for two types of silicon diode detectors. For 6‐MV x rays, the CFs for the ISORAD diode are 0.96 at TSD of 70 cm and 1.02 at TSD of 130 cm, while CFs for the QED diode are within ±1% from 1.00 for the same range of TSD. For 18‐MV x rays, the CFs for the ISORAD diode are 0.94 at TSD of 70 cm and 1.02 at TSD of 130 cm while CFs for the QED diode are 0.97 at TSD of 70 cm and 1.01 at TSD of 130 cm.

**Table I:** Comparison TSD correction factors for two types of diode detectors measured with the field size of $10\times 10\;{\text{cm}}^{2}$.

	6‐MV CF		18‐MV CF	
TSD (cm)	QED	ISORAD	QED	ISORAD
70	0.99	0.96	0.97	0.94
80	1.00	0.98	0.99	0.97
90	1.00	0.99	1.00	1.00
100	1.00	1.00	1.00	1.00
110	1.00	1.00	1.00	1.01
120	1.01	1.02	1.00	1.02
130	1.01	1.02	1.01	1.02

### Field size dependence

**Figure 1:**
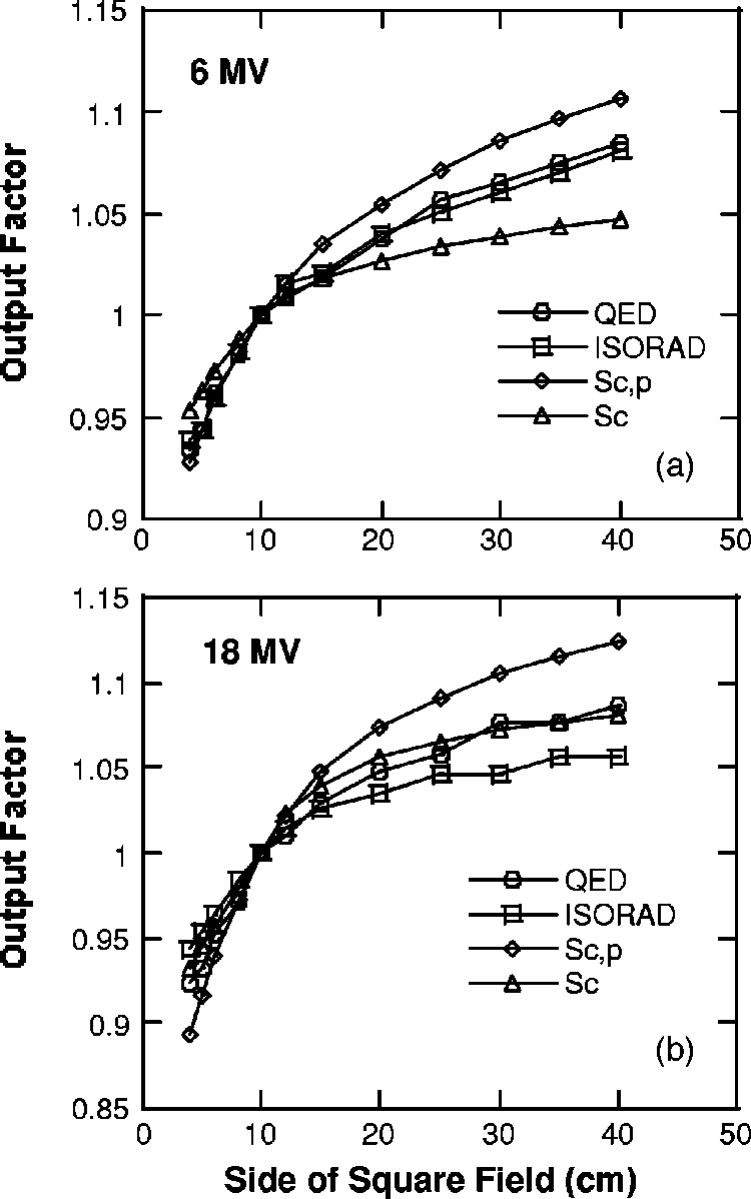
Comparison of output factors for two types of diode detectors as a function of square field size (a) for the 6‐MV beam and (b) for the 18‐MV beam. Also included are total scatter factors and collimator scatter factors of both beams.

Figure 1 shows output factors of diode detectors [defined in Eq. (1)] as a function of side of the square field. Also included in Fig. 1 are total scatter factors, $S_{c,p}$, and collimator scatter factors, $S_{c}$. For 6‐MV x rays, the diode output factors for ISORAD and QED diodes are very similar for the entire range of field sizes considered here. On the other hand, diode output factors are quite different from either total scatter factors or collimator scatter factors, especially for field sizes larger than $10\times 10\;{\text{cm}}^{2}$. For 18‐MV x rays, diode output factors for the QED diode is comparable with collimator scatter factors, not total scatter factors, except for very small field sizes, while diode output factors for the ISORAD diode do not resemble to either total scatter factors or collimator scatter factors. Table II lists the field size correction factors derived from the data in Fig. 1, as defined in Eq. (3). For the 6‐MV beam, both ISORAD and QED have similar correction factors, within 2% from 1, for the entire field size range studied here. For the 18‐MV beam, the CFs for the ISORAD diode are 0.95 for the field size of $4\times 4\;{\text{cm}}^{2}$ and 1.06 for the field size $40\times 40\;{\text{cm}}^{2}$, while the CFs for the QED diode are 0.97 for the field size of $4\times 4\;{\text{cm}}^{2}$ and 1.04 for the field size of $40\times 40\;{\text{cm}}^{2}$.

**Table II:** Comparison field size correction factors for two types of diode detectors measured at TSD of 100 cm.

	6‐MVCF		18‐MV CF	
Field size (cm×cm)	QED	ISORAD	QED	ISORAD
4×4	0.99	0.99	0.97	0.95
5×5	1.00	1.00	0.98	0.96
6×6	1.00	1.00	0.99	0.97
8×8	1.00	1.00	1.00	0.99
10×10	1.00	1.00	1.00	1.00
12×12	1.01	1.00	1.01	1.01
15×15	1.02	1.01	1.02	1.02
20×20	1.02	1.01	1.03	1.04
25×25	1.02	1.02	1.03	1.04
30×30	1.02	1.02	1.03	1.06
40×40	1.02	1.02	1.04	1.06

### Wedge correction factors

Table III lists the measured correction factors for wedges. Wedge CFs for ISORAD diodes are greater than that for QED especially for 45° and 60° wedges. In fact, for the 60° wedge, CFs for ISORAD dosimeters are 1.07 for the 6‐MV beam and 1.05 for the 18‐MV beam. In contrast, for 18‐MV x rays, CFs for the QED diode for all wedges are within 1% from 1; for 6 MV, the only significant CF is for the 60° wedge, which is equal to 1.03.

**Table III:** Comparison wedge correction factors for two types of diode detectors measured with the field size of $10\times 10\;{\text{cm}}^{2}$.

	6‐MV CF		18‐MV CF	
Wedge	QED	ISORAD	QED	ISORAD
15°	1.01	1.02	1.01	1.00
30°	1.01	1.03	1.00	1.01
45°	1.02	1.06	1.01	1.04
60°	1.03	1.07	1.01	1.05

## DISCUSSION

In general, the entrance dose, $D_{\text{entrance}}$ is not equal to diode readout even if it is an ideal diode detector and no correction factor is needed. The calibration factor, $F_{\text{calib}}$, which may deviate significantly from 1, depending on the calibration setups for both photon beams itself and the diode, is the proportional factor relating diode readout to $D_{\text{entrance}}$. Both types of diode detectors studied here are not ideal. Therefore, correction factors are needed to accurately predict $D_{\text{entrance}}$ from diode readout. In general, QED (*p*‐type) diodes require smaller correction factors.

For QED dosimeters, our correction factors for TSD are consistent with the vendor's specifications. The values are also consistent with published data for *p*‐type silicon diodes.^10^ ^,^ ^11^ It is well known that diode detectors deviate from the inverse square law, especially for short TSDs. This has been attributed to the instantaneous dose rate dependence of diode detectors.^15^ This instantaneous dose rate dependence is one of the effects of radiation damage. Radiation damage has been shown to be more serious for *n*‐type than for *p*‐type detectors.^16^ ^–^ ^19^ The radiation damage for *p*‐type detectors can be significantly reduced after being pre‐irradiated to 10 kGy or more with high energy electron beams.^18^ The QED diode detectors were pre‐irradiated to 10 kGy with a 10‐MeV electron beam according to the vendor's data sheet. This is consistent with our observation that QED detectors have smaller TSD CFs than ISORAD detectors.

Differences in diode output factors and total scatter factors measured by an ion chamber mandate the need for correction factors for field size dependence. It is expected that there are some differences between diode output factors and total scatter factors measured by an ion chamber in phantom, considering the fact that the diode is placed at the phantom surface and an ion chamber is immersed in the phantom at $d_{\max}$ resulting in different scatter conditions. It is also not surprising to observe differences between diode output factors and collimator scatter factors because different buildup materials and setups were used. The observed coincidence between ${\text{OF}}_{\text{diode}}$ for the 18‐MV QED diode with collimator scatter factors might be due to the fact that both had approximately $3.0\;g/{\text{cm}}^{2}$ buildup materials. In general, however, we should not assume that collimator scatter factors could be used in place of diode output factors.^13^

Instantaneous dose rate dependence also manifests in wedge correction factors.^15^ Wedges in general will reduce the instantaneous dose rate roughly by a factor of the wedge factor. ISORAD detectors have larger correction factors than QED detectors. In fact, for 18‐MV x rays, wedge correction factors for the QED detector are practically 1. This is again consistent with the theory of radiation damage for *n*‐type and *p*‐type diodes. Beam hardening also contributes to the wedge correction factors.^6^ It is well known that the effect of beam hardening is more profound for 6‐MV x‐ray beams than 18‐MV beams.

We have also studied the effect of block trays on diode and found the correction factor to be within 1.000±0.005. Therefore, we have decided to not use a block tray correction factor for our diode dosimetry program. In addition to correction factors studied here, if compensator filters are used, it is necessary to determine correction factors for compensator filters as a part of commission efforts of a diode based *in‐vivo* dosimetry program.

Temperature can affect the diode response.^6^ ^,^ ^20^ ^,^ ^21^ Grusell and Rikner found increased sensitivity for *p*‐type detectors with increasing temperature, especially after an accumulated dose.^20^ Van Dam, Leunens, and Detreix later confirmed this temperature effect, but decided clinically not to correct for the temperature effect, except for long irradiation such as TBI at low dose rate.^21^ They argued that the time between taping of the detector and start of the patient irradiation was very short (about 10 s) and that the treatment times were on the order of 1 min. Furthermore, Heukelom, Lanson, and Mijnheer found that several diodes they studied can be divided into three groups with respect to their response to temperature, namely, an increase, a decrease and a constant response.^6^ They suggested that further investigation of the temperature properties of all diodes was required to explain the three different temperature responses. Based on these studies,^6^ ^,^ ^20^ ^,^ ^21^ we have not included the temperature effect for typical clinical situations in our program.

## CONCLUSION

We have studied TSD, field size and wedge dependences of two types of commercial diode detectors, ISORAD (*n*‐type) and QED (*p*‐type), for *in‐vivo* dosimetry. A formula is provided to relate diode readings to entrance doses. In this formula, correction factors for TSD, field size, and wedges are defined. Correction factors are derived from measured data. Our results are consistent with the theory of radiation damage of silicon diodes. We have found that both types of diodes are not ideal and require correction factors. In general, correction factors for QED (*p*‐type) diodes are smaller than ISORAD (*n*‐type) detectors. Correction factors reported here for either type of diode should not be used without verification.

## ACKNOWLEDGMENTS

I would like to thank Jie Shi of Sun Nuclear Corp. for interesting discussions about silicon diode detectors.

## References

[1] G. H. Fletcher , Textbook of Radiation Therapy, 3rd ed. (Lea and Febiger, Philadalphia, 1981).

[2] M. Essers and B. J. Mijnheer , “In vivo dosimetry during external beam photon beam radiotherapy,” Int. J. Radiat. Oncol., Biol., Phys. 43, 245–259 (1999). 10030247 10.1016/s0360-3016(98)00341-1

[3] R. Alecu , M. Alecu , and T. G. Ochran , “A method to improve the effectiveness of diode in vivo dosimetry,” Med. Phys. 25, 746–749 (1998). 9608486 10.1118/1.598237

[4] D. P. Fontenla , J. Curran , R. Yaparpalvi , and B. Vikram , “Customization of a radiation management system to support in vivo patient dosimetry using diodes,” Med. Phys. 23, 1425–1429 (1996). 8873041 10.1118/1.597744

[5] D. P. Fontenla , R. Yaparpalvi , C.‐S. Chui , and E. Briot , “The use of diode dosimetry in quality improvement of patient care in radiation therapy,” Med. Dosim 21, 235–241 (1996). 8985929 10.1016/s0958-3947(96)00081-7

[6] S. Heukelom , J. H. Lanson , and B. J. Mijnheer , “Comparison of entrance and exit dose measurements using ionization chambers and silicon diodes,” Phys. Med. Biol. 36, 47–59 (1991). 2006214 10.1088/0031-9155/36/1/005

[7] P. Lee , J. M. Sawicka , and G. P. Glasgow , “Patient dosimetry quality assurance program with a commercial diode system,” Int. J. Radiat. Oncol., Biol., Phys. 29, 1175–1182 (1994). 8083088 10.1016/0360-3016(94)90415-4

[8] G. Leunens , J. Van Dam , A. Dutreix , and E. van der Schueren , “Quality assurance in radiotherapy by in vivo dosimetry. 1. Entrance dose measurements, a reliable procedure,” Radiother. Oncol. 17, 141–151 (1990). 2320746 10.1016/0167-8140(90)90102-3

[9] G. Leunens , J. Van Dam , A. Dutreix , and E. van der Schueren , “Quality assurance in radiotherapy by in vivo dosimetry. 2. Determination of the target absorbed dose,” Radiother. Oncol. 19, 73–87 (1990). 2236639 10.1016/0167-8140(90)90167-u

[10] Th. Loncol , J. L. Greffe , S. Vynckier , and P. Scalliet , “Entrance and exit dose measurements with semiconductors and thermoluminescent dosimeters: a comparison of methods and in vivo results,” Radiother. Oncol. 41, 179–187 (1996). 9004362 10.1016/s0167-8140(96)01826-9

[11] R. W. Luse , J. Eenmaa , T. Kwiatkowski , and D. Schumacher , “In vivo diode dosimetry for total marrow irradiation,” Int. J. Radiat. Oncol., Biol., Phys. 36, 189–195 (1996). 8823275 10.1016/0360-3016(95)02105-1

[12] R. J. Meiler and M. B. Podgorsak , “Characterization of the response of commercial diode detectors used for in vivo dosimetry,” Med. Dosim 22, 31–37 (1997). 9136105 10.1016/s0958-3947(96)00152-5

[13] J. G. Wierzbicki and D. S. Waid , “Large discrepancies between calculated $D_{\max}$ and diode readings for small field size and small SSDs of 15 MV photon beams,” Med. Phys. 25, 245–246 (1998). 9507487 10.1118/1.598192

[14] Task Group 21, Radiation Therapy Committee, American Association of Physicists in Medicine , “A protocol for the determination of absorbed dose from high‐energy photon and electron beams,” Med. Phys. 10, 741–771 (1983). 6419029 10.1118/1.595446

[15] J. Shi , W. E. Simon , T. C. Zhu , and A. Saini , “Theoretical model for the SSD dependence of Si diode detectors in the application of dosimetry,” Med. Phys. 23, 1072 (1996) (Abstract).

[16] G. Rikner and E. Grusell , “Effects of radiation damage on p‐type silicon detectors,” Phys. Med. Biol. 28, 1261–1267 (1983).

[17] G. Rikner and E. Grusell , “General specifications for silicon semiconductors for use in radiation dosimetry,” Phys. Med. Biol. 32, 1109–1117 (1987). 3671497 10.1088/0031-9155/32/9/004

[18] E. Grusell and G. Rikner , “Radiation damage induced dose rate non‐linearity in an n‐type silicon detector,” Acta Radiol.: Oncol. 23, 456–459 (1984). 10.3109/02841868409136050 6099041

[19] E. Grusell and G. Rikner , “Linearity with dose rate of low resistivity p‐type silicon semiconductor detectors,” Phys. Med. Biol. 38, 785–792 (1993).

[20] E. Grusell and G. Rikner , “Evaluation of temperature effects in p‐type silicon diode,” Phys. Med. Biol. 31, 527–534 (1986).

[21] J. Van Dam , G. Leunens , and A. Dutreix , “Correlation between temperature and dose rate dependence of semiconductor response: influence of accumulated dose,” Radiother. Oncol. 19, 345–351 (1990). 2284444 10.1016/0167-8140(90)90035-u

